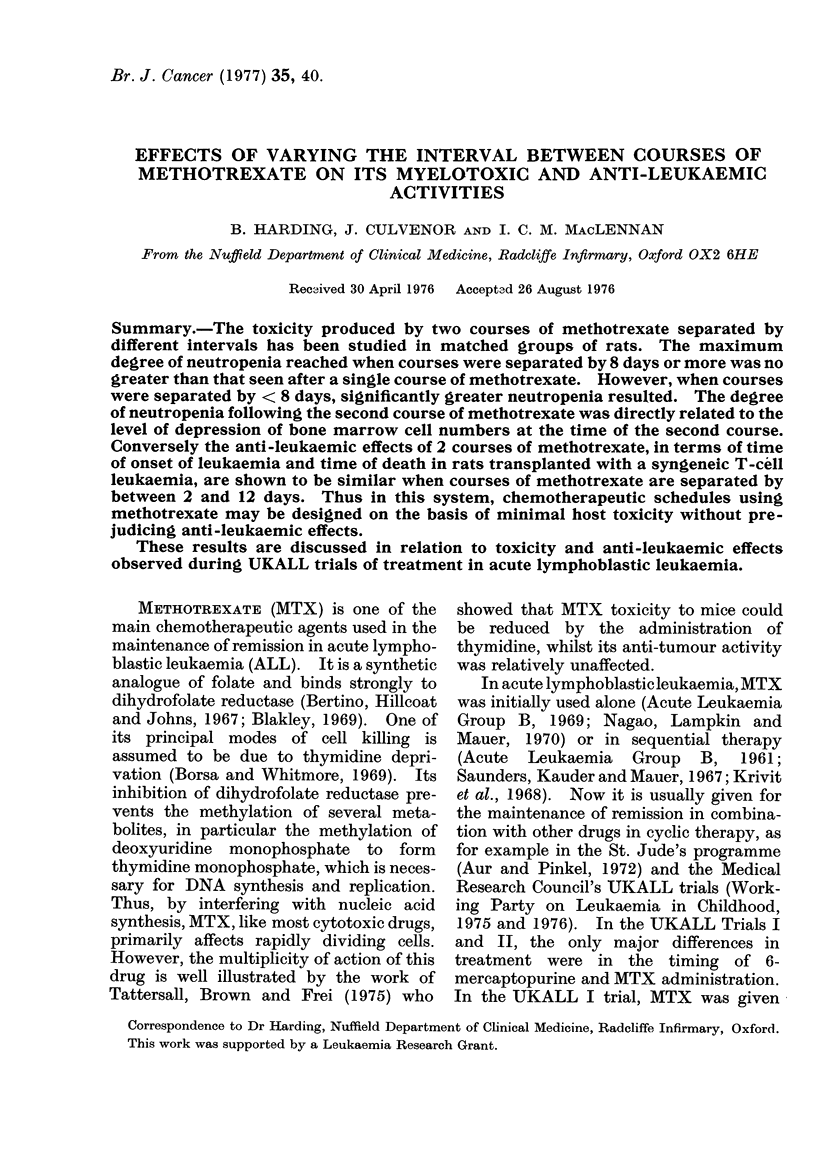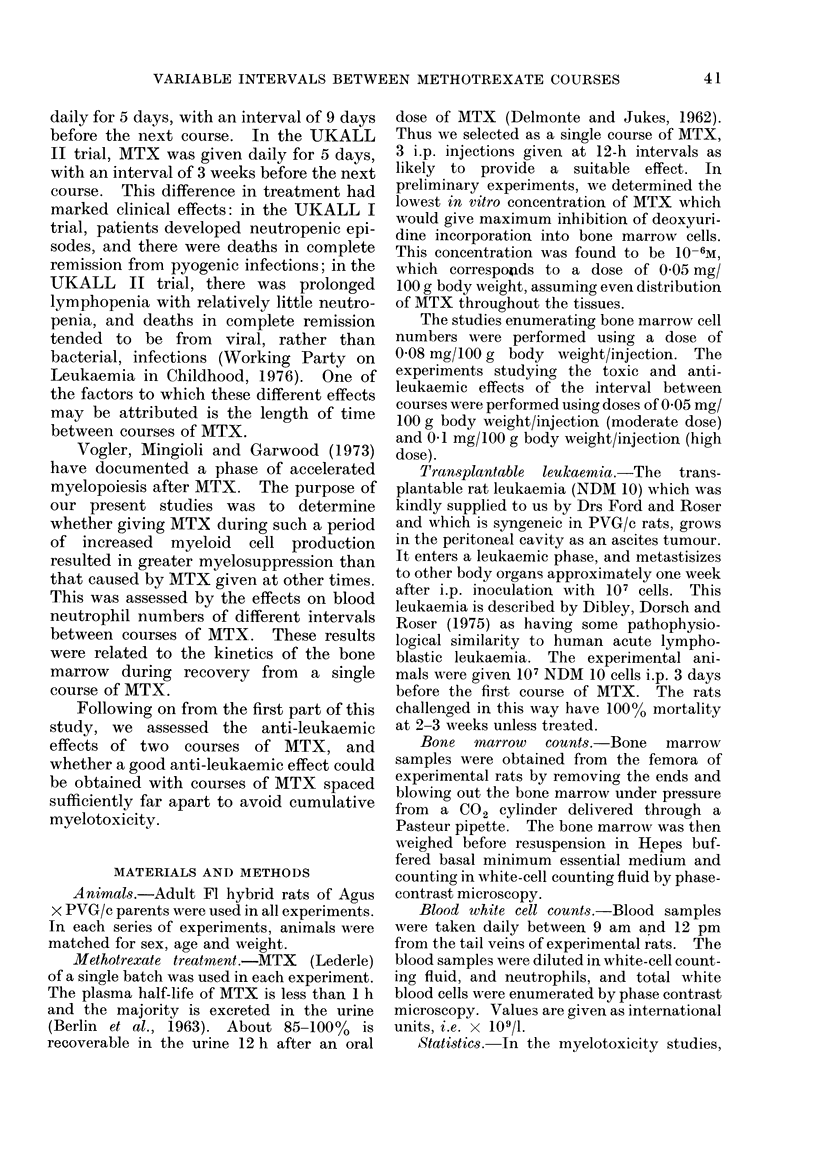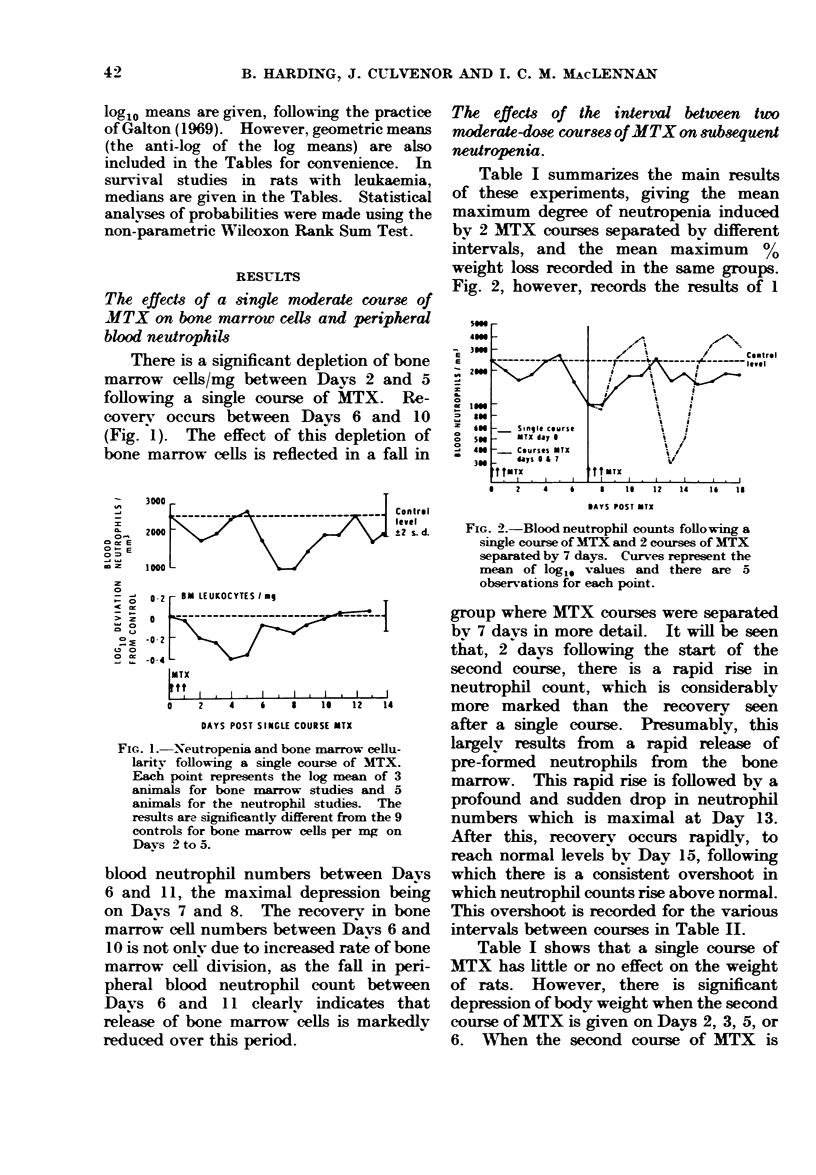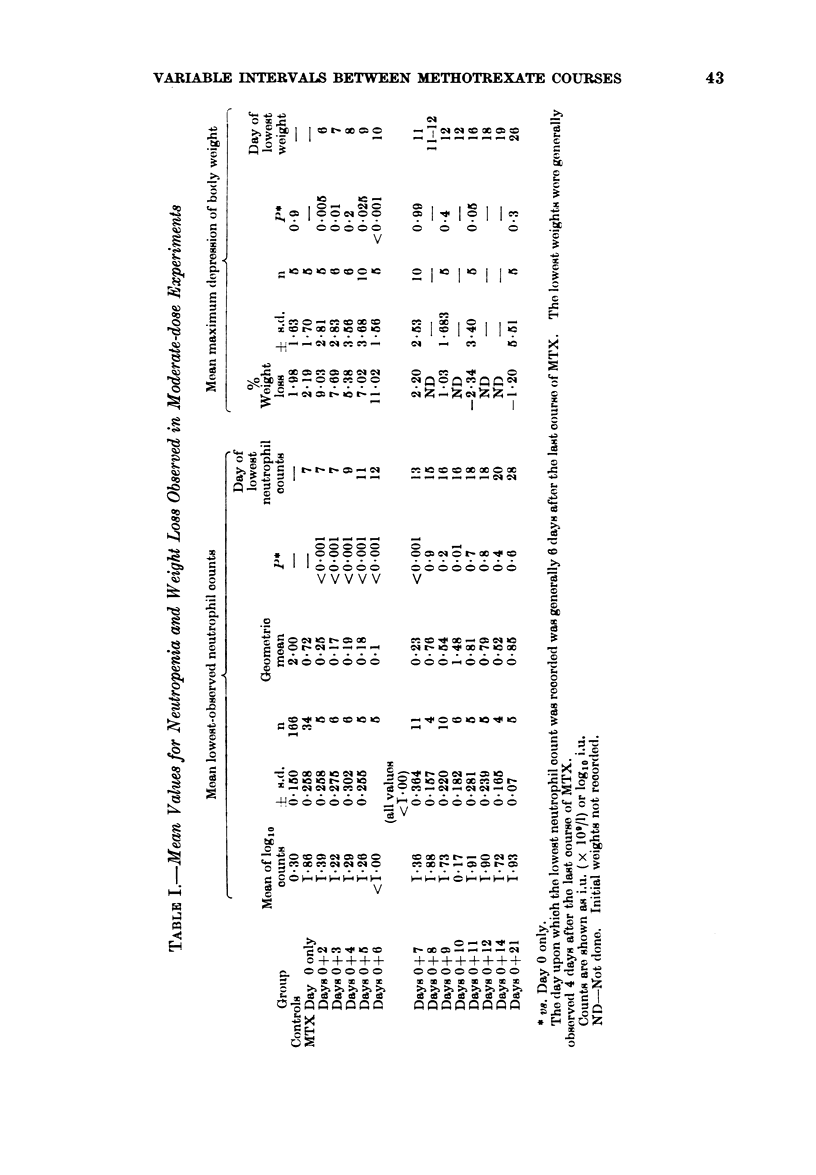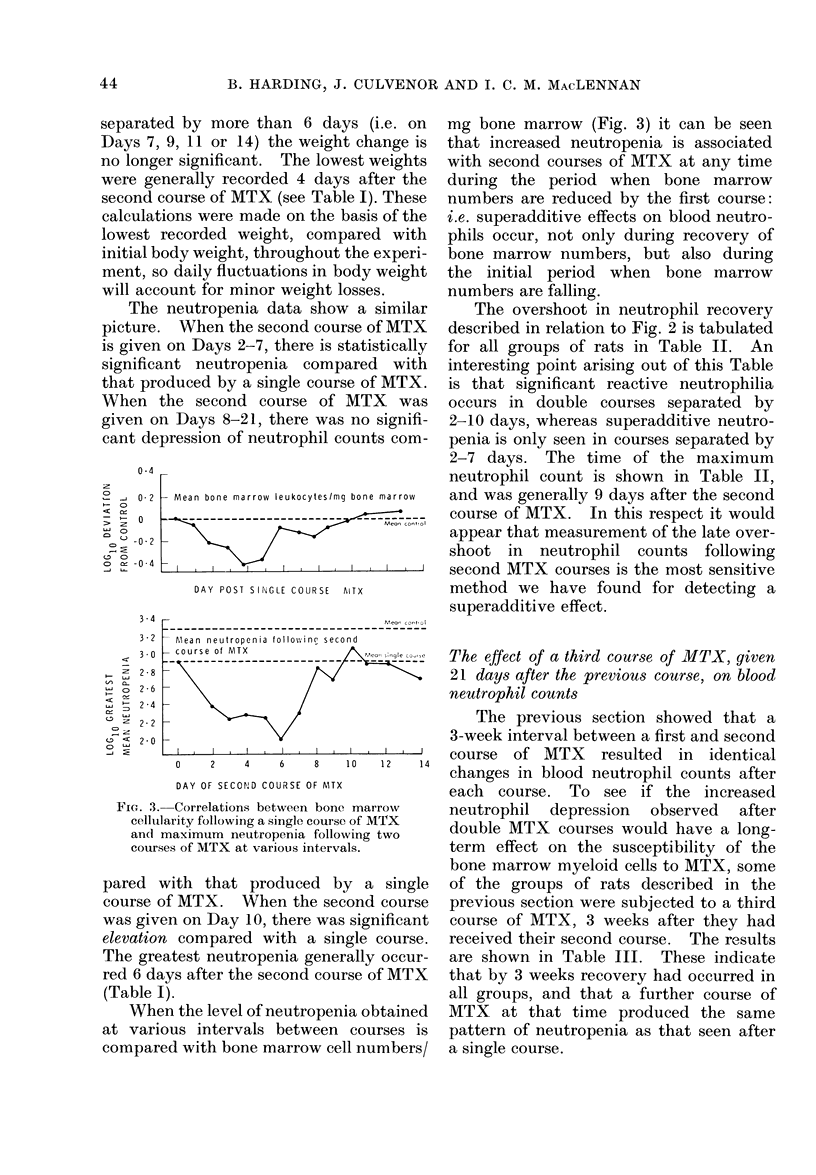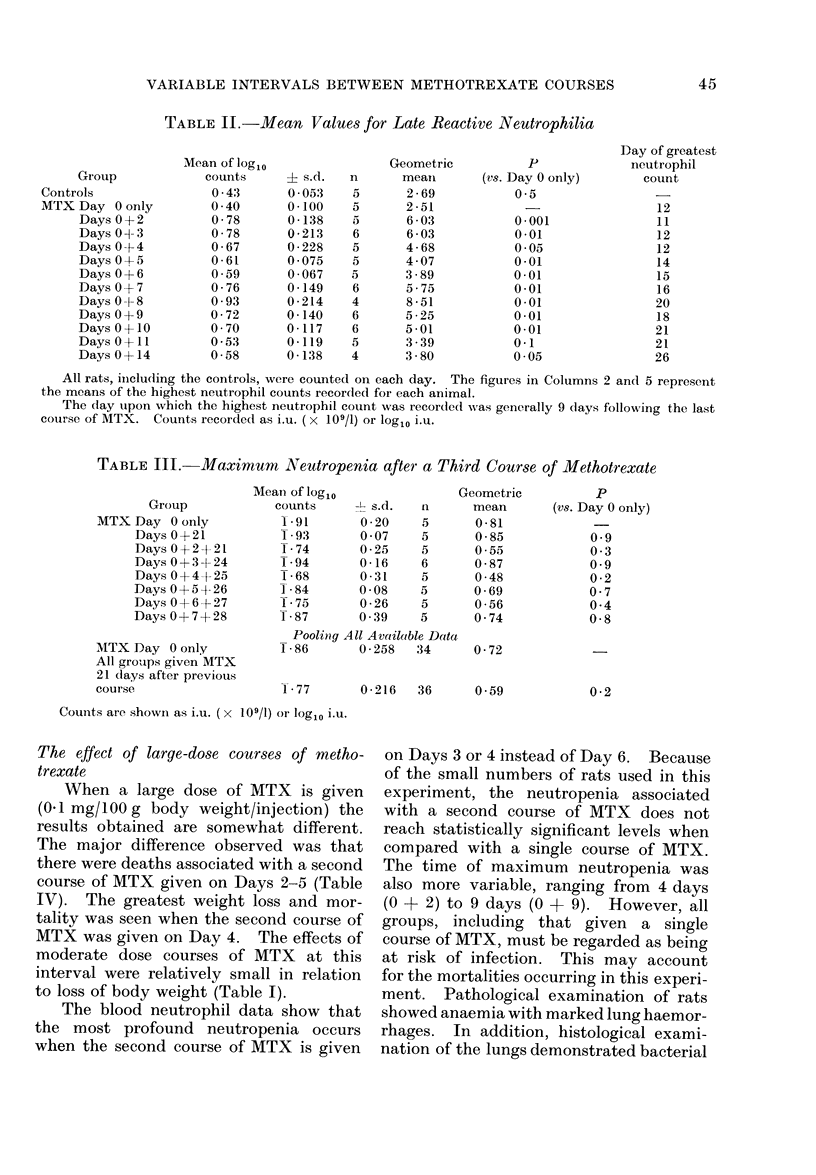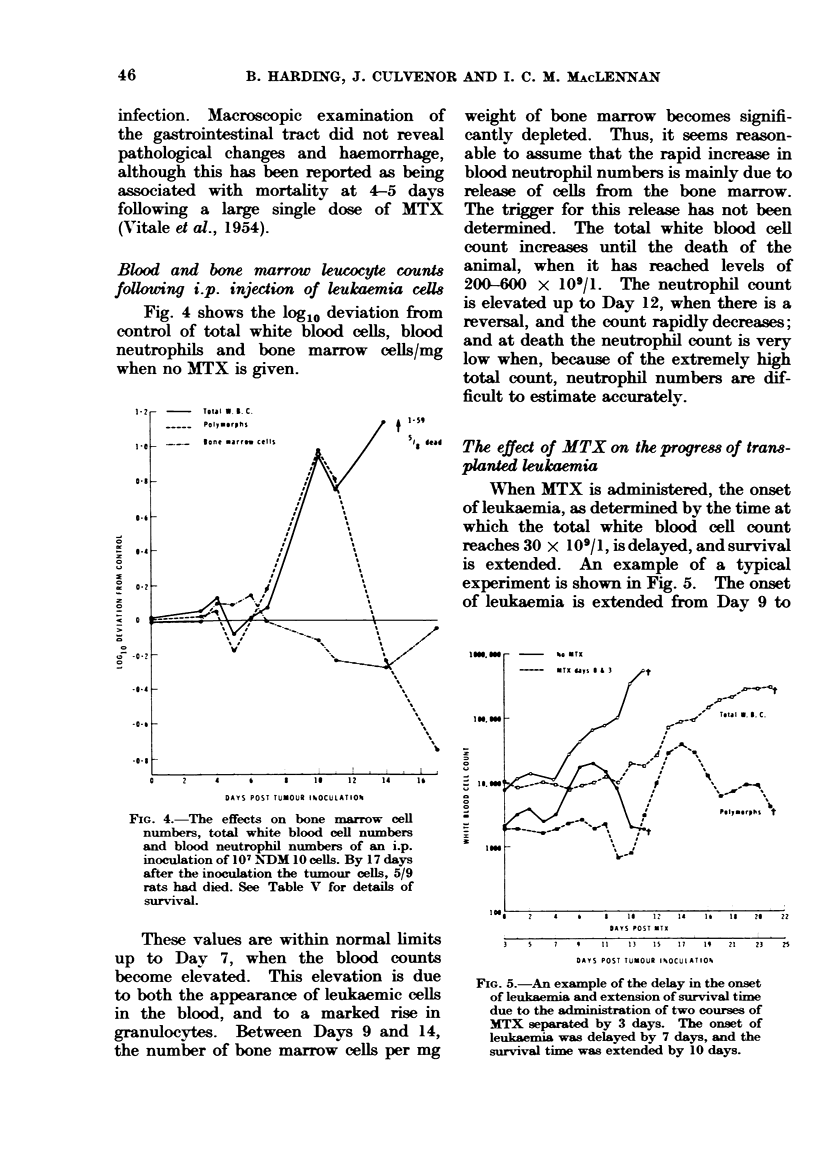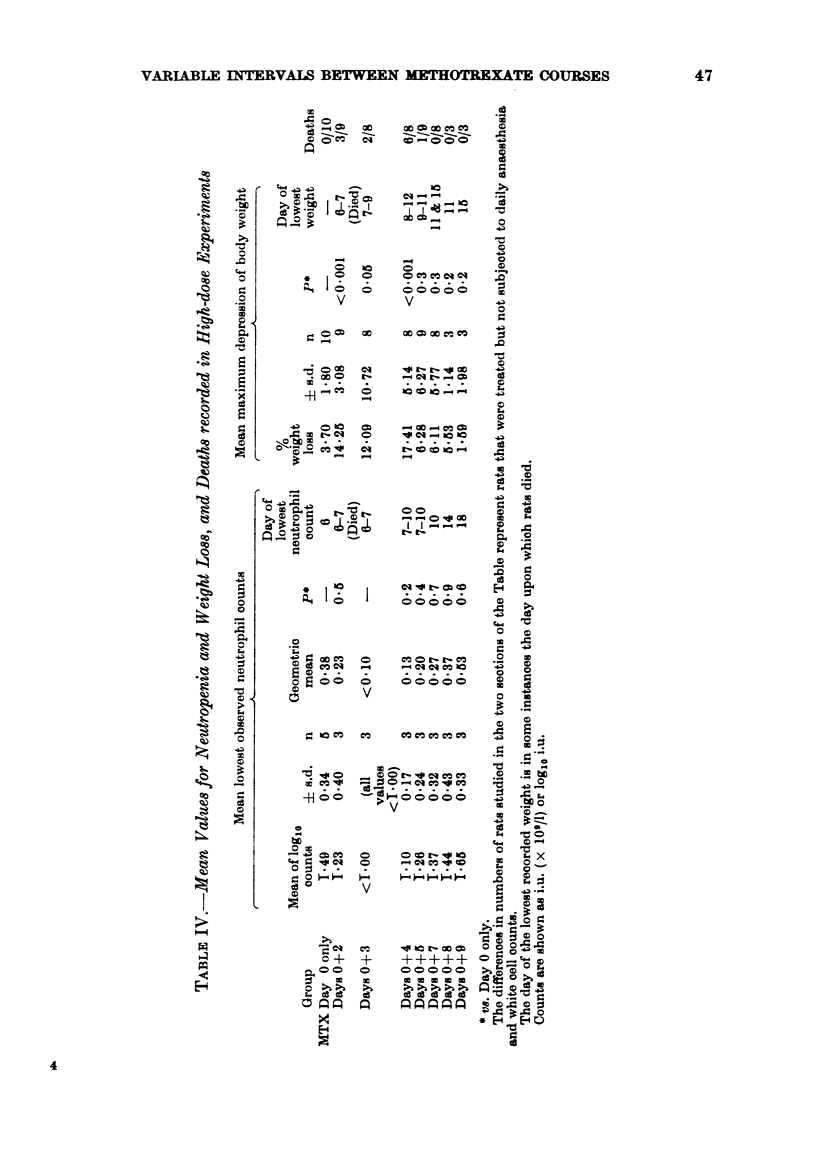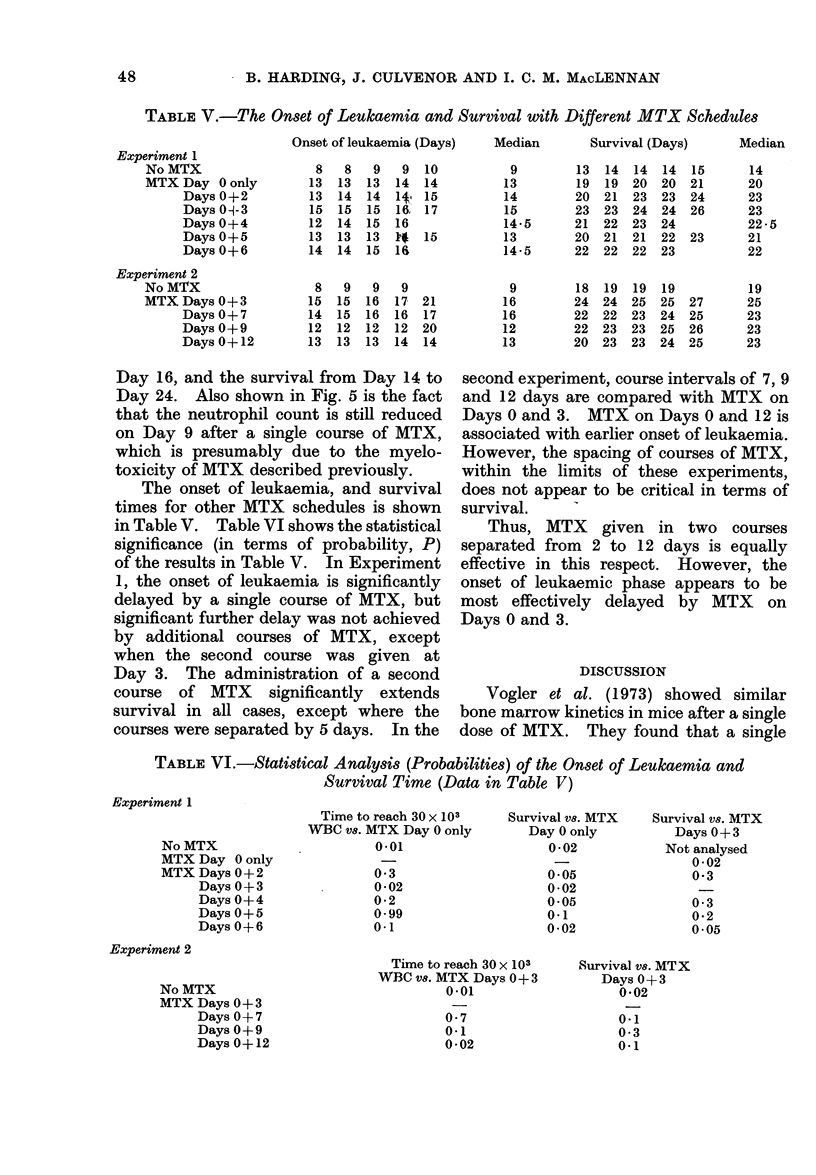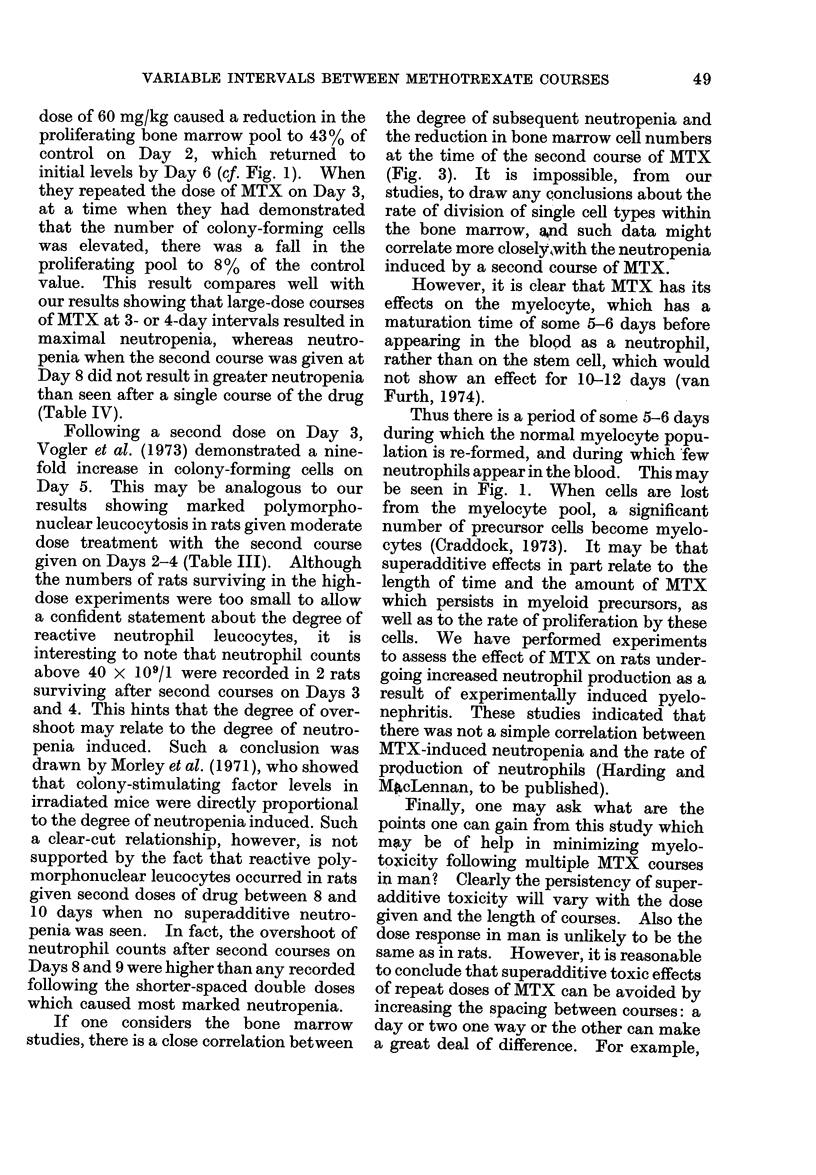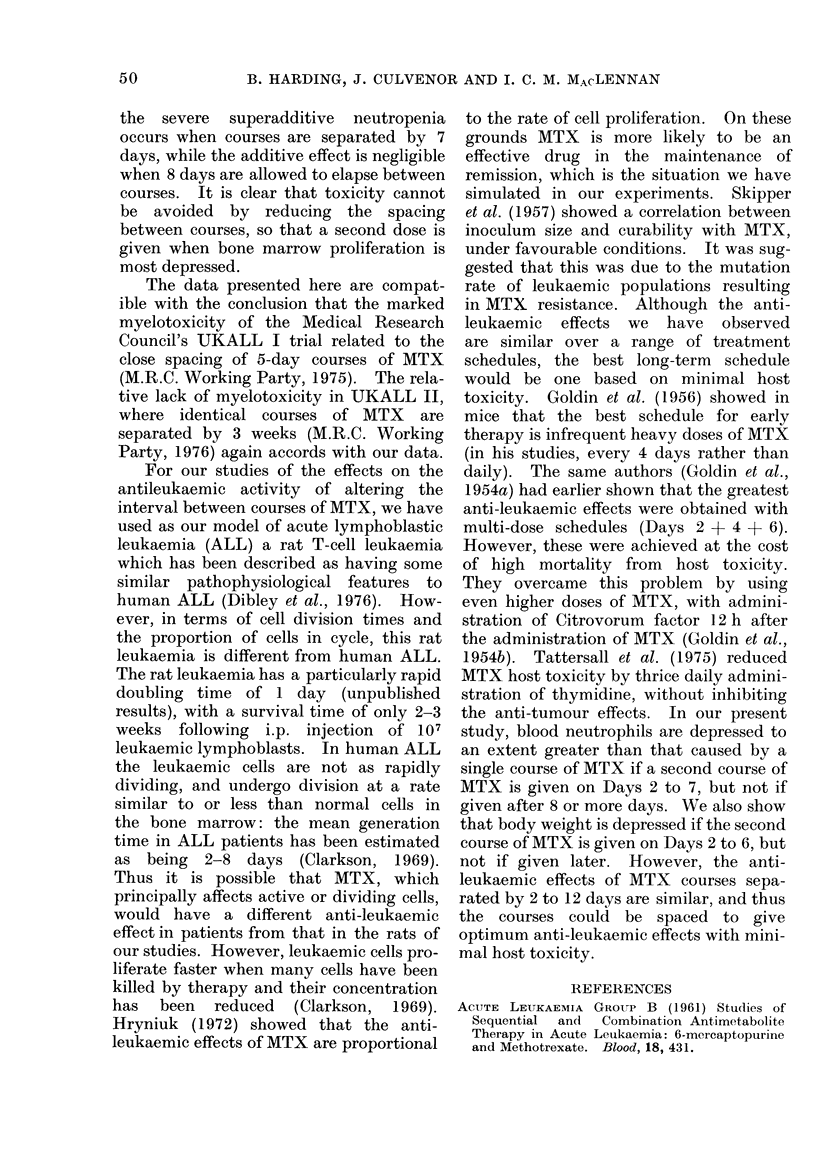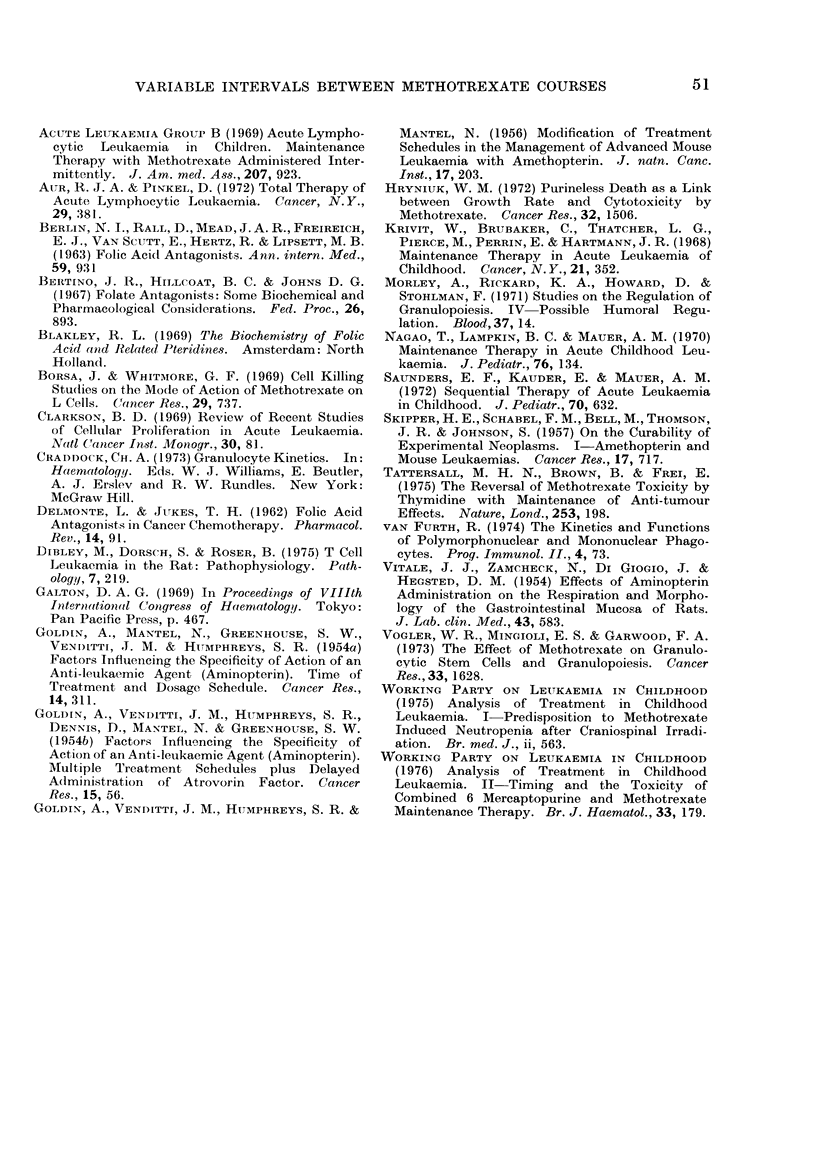# Effects of varying the interval between courses of methotrexate on its myelotoxic and anti-leukaemic activities.

**DOI:** 10.1038/bjc.1977.2

**Published:** 1977-01

**Authors:** B. Harding, J. Culvenor, I. C. MacLennan

## Abstract

The toxicity produced by two courses of methotrexate separated by different intervals has been studied in matched groups of rats. The maximum degree of neutropenia reached when courses were separated by 8 days or more was no greater than that seen after a single course of methotrexate. However, when courses of neutropenia following the second course of methotrexate was directly related to the level of depression of bone marrow cell numbers at the time of the second course. Conversely the anti-leukaemic effects of 2 courses of methotrexate, in terms of time of onset of leukaemia and time of death in rats transplanted with a syngeneic T-cell leukaemia, are shown to be similar when courses of methotrexate are separated by between 2 and 12 days. Thus in this system, chemotherapeutic schedules using methotrexate may be designed on the basis of minimal host toxicity without prejudicing anti-leukaemic effects. These results are discussed in relation to toxicity and anti-leukaemic effects observed during UKALL trials of treatment in acute lymphoblastic leukaemia.


Br. J. Cancer (1977) 35, 40.

### EFFECTS OF VARYING THE INTERVAL BETWEEN COURSES OF METHOTREXATE ON ITS MYELOTOXIC AND ANTI-LEUKAEMIC ACTIVITIES

B. HARDING, J. CULVENOR AND I. C. M. MACLENNAN

*From the Nuffield Department of Clinical Medicine, Radcliffe Infirmary, Oxford OX2 6HE*

Received 30 April 1976 Accepted 26 August 1976

**Summary.—The toxicity produced by two courses of methotrexate separated by different intervals has been studied in matched groups of rats. The maximum degree of neutropenia reached when courses were separated by 8 days or more was no greater than that seen after a single course of methotrexate. However, when courses were separated by < 8 days, significantly greater neutropenia resulted. The degree of neutropenia following the second course of methotrexate was directly related to the level of depression of bone marrow cell numbers at the time of the second course. Conversely the anti-leukaemic effects of 2 courses of methotrexate, in terms of time of onset of leukaemia and time of death in rats transplanted with a syngeneic T-cell leukaemia, are shown to be similar when courses of methotrexate are separated by between 2 and 12 days. Thus in this system, chemotherapeutic schedules using methotrexate may be designed on the basis of minimal host toxicity without prejudicing anti-leukaemic effects.**

**These results are discussed in relation to toxicity and anti-leukaemic effects observed during UKALL trials of treatment in acute lymphoblastic leukaemia.**

METHOTREXATE (MTX) is one of the main chemotherapeutic agents used in the maintenance of remission in acute lymphoblastic leukaemia (ALL). It is a synthetic analogue of folate and binds strongly to dihydrofolate reductase (Bertino, Hillcoat and Johns, 1967; Blakley, 1969). One of its principal modes of cell killing is assumed to be due to thymidine deprivation (Borsa and Whitmore, 1969). Its inhibition of dihydrofolate reductase prevents the methylation of several metabolites, in particular the methylation of deoxyuridine monophosphate to form thymidine monophosphate, which is necessary for DNA synthesis and replication. Thus, by interfering with nucleic acid synthesis, MTX, like most cytotoxic drugs, primarily affects rapidly dividing cells. However, the multiplicity of action of this drug is well illustrated by the work of Tattersall, Brown and Frei (1975) who showed that MTX toxicity to mice could be reduced by the administration of thymidine, whilst its anti-tumour activity was relatively unaffected.

In acute lymphoblastic leukaemia, MTX was initially used alone (Acute Leukaemia Group B, 1969; Nagao, Lampkin and Mauer, 1970) or in sequential therapy (Acute Leukaemia Group B, 1961; Saunders, Kauder and Mauer, 1967; Krivit *et al.*, 1968). Now it is usually given for the maintenance of remission in combination with other drugs in cyclic therapy, as for example in the St. Jude's programme (Aur and Pinkel, 1972) and the Medical Research Council's UKALL trials (Working Party on Leukaemia in Childhood, 1975 and 1976). In the UKALL Trials I and II, the only major differences in treatment were in the timing of 6-mercaptopurine and MTX administration. In the UKALL I trial, MTX was given

Correspondence to Dr Harding, Nuffield Department of Clinical Medicine, Radcliffe Infirmary, Oxford.
This work was supported by a Leukaemia Research Grant.

daily for 5 days, with an interval of 9 days before the next course. In the UKALL II trial, MTX was given daily for 5 days, with an interval of 3 weeks before the next course. This difference in treatment had marked clinical effects: in the UKALL I trial, patients developed neutropenic episodes, and there were deaths in complete remission from pyogenic infections; in the UKALL II trial, there was prolonged lymphopenia with relatively little neutropenia, and deaths in complete remission tended to be from viral, rather than bacterial, infections (Working Party on Leukaemia in Childhood, 1976). One of the factors to which these different effects may be attributed is the length of time between courses of MTX.

Vogler, Mingioli and Garwood (1973) have documented a phase of accelerated myelopoiesis after MTX. The purpose of our present studies was to determine whether giving MTX during such a period of increased myeloid cell production resulted in greater myelosuppression than that caused by MTX given at other times. This was assessed by the effects on blood neutrophil numbers of different intervals between courses of MTX. These results were related to the kinetics of the bone marrow during recovery from a single course of MTX.

Following on from the first part of this study, we assessed the anti-leukaemic effects of two courses of MTX, and whether a good anti-leukaemic effect could be obtained with courses of MTX spaced sufficiently far apart to avoid cumulative myelotoxicity.

### MATERIALS AND METHODS

*Animals.*—Adult Fl hybrid rats of Agus × PVG/c parents were used in all experiments. In each series of experiments, animals were matched for sex, age and weight.

*Methotrexate treatment.*—MTX (Lederle) of a single batch was used in each experiment. The plasma half-life of MTX is less than 1 h and the majority is excreted in the urine (Berlin *et al.*, 1963). About 85–100% is recoverable in the urine 12 h after an oral dose of MTX (Delmonte and Jukes, 1962). Thus we selected as a single course of MTX, 3 i.p. injections given at 12-h intervals as likely to provide a suitable effect. In preliminary experiments, we determined the lowest *in vitro* concentration of MTX which would give maximum inhibition of deoxyuridine incorporation into bone marrow cells. This concentration was found to be $10^{-6}$M, which corresponds to a dose of 0·05 mg/100 g body weight, assuming even distribution of MTX throughout the tissues.

The studies enumerating bone marrow cell numbers were performed using a dose of 0·08 mg/100 g body weight/injection. The experiments studying the toxic and anti-leukaemic effects of the interval between courses were performed using doses of 0·05 mg/100 g body weight/injection (moderate dose) and 0·1 mg/100 g body weight/injection (high dose).

*Transplantable leukaemia.*—The transplantable rat leukaemia (NDM 10) which was kindly supplied to us by Drs Ford and Roser and which is syngeneic in PVG/c rats, grows in the peritoneal cavity as an ascites tumour. It enters a leukaemic phase, and metastisizes to other body organs approximately one week after i.p. inoculation with $10^7$ cells. This leukaemia is described by Dibley, Dorsch and Roser (1975) as having some pathophysiological similarity to human acute lymphoblastic leukaemia. The experimental animals were given $10^7$ NDM 10 cells i.p. 3 days before the first course of MTX. The rats challenged in this way have 100% mortality at 2–3 weeks unless treated.

*Bone marrow counts.*—Bone marrow samples were obtained from the femora of experimental rats by removing the ends and blowing out the bone marrow under pressure from a $CO_2$ cylinder delivered through a Pasteur pipette. The bone marrow was then weighed before resuspension in Hepes buffered basal minimum essential medium and counting in white-cell counting fluid by phase-contrast microscopy.

*Blood white cell counts.*—Blood samples were taken daily between 9 am and 12 pm from the tail veins of experimental rats. The blood samples were diluted in white-cell counting fluid, and neutrophils, and total white blood cells were enumerated by phase contrast microscopy. Values are given as international units, *i.e.* $\times 10^9$/l.

*Statistics.*—In the myelotoxicity studies,

$\log_{10}$ means are given, following the practice of Galton (1969). However, geometric means (the anti-log of the log means) are also included in the Tables for convenience. In survival studies in rats with leukaemia, medians are given in the Tables. Statistical analyses of probabilities were made using the non-parametric Wilcoxon Rank Sum Test.

### RESULTS

### *The effects of a single moderate course of MTX on bone marrow cells and peripheral blood neutrophils*

There is a significant depletion of bone marrow cells/mg between Days 2 and 5 following a single course of MTX. Recovery occurs between Days 6 and 10 (Fig. 1). The effect of this depletion of bone marrow cells is reflected in a fall in

FIG. 1.—Neutropenia and bone marrow cellularity following a single course of MTX. Each point represents the log mean of 3 animals for bone marrow studies and 5 animals for the neutrophil studies. The results are significantly different from the 9 controls for bone marrow cells per mg on Days 2 to 5.

blood neutrophil numbers between Days 6 and 11, the maximal depression being on Days 7 and 8. The recovery in bone marrow cell numbers between Days 6 and 10 is not only due to increased rate of bone marrow cell division, as the fall in peripheral blood neutrophil count between Days 6 and 11 clearly indicates that release of bone marrow cells is markedly reduced over this period.

### *The effects of the interval between two moderate-dose courses of MTX on subsequent neutropenia.*

Table I summarizes the main results of these experiments, giving the mean maximum degree of neutropenia induced by 2 MTX courses separated by different intervals, and the mean maximum % weight loss recorded in the same groups. Fig. 2, however, records the results of 1

FIG. 2.—Blood neutrophil counts following a single course of MTX and 2 courses of MTX separated by 7 days. Curves represent the mean of $\log_{10}$ values and there are 5 observations for each point.

group where MTX courses were separated by 7 days in more detail. It will be seen that, 2 days following the start of the second course, there is a rapid rise in neutrophil count, which is considerably more marked than the recovery seen after a single course. Presumably, this largely results from a rapid release of pre-formed neutrophils from the bone marrow. This rapid rise is followed by a profound and sudden drop in neutrophil numbers which is maximal at Day 13. After this, recovery occurs rapidly, to reach normal levels by Day 15, following which there is a consistent overshoot in which neutrophil counts rise above normal. This overshoot is recorded for the various intervals between courses in Table II.

Table I shows that a single course of MTX has little or no effect on the weight of rats. However, there is significant depression of body weight when the second course of MTX is given on Days 2, 3, 5, or 6. When the second course of MTX is

TABLE I.—*Mean Values for Neutropenia and Weight Loss Observed in Moderate-dose Experiments*

| | Mean lowest-observed neutrophil counts | | | | | | Mean maximum depression of body weight | | | | |
|---|---|---|---|---|---|---|---|---|---|---|---|
| Group | Mean of $\log_{10}$ counts | ± s.d. | n | Geometric mean | $P$* | Day of lowest neutrophil counts | % Weight loss | ± s.d. | n | $P$* | Day of lowest weight |
| Controls | 0·30 | 0·150 | 166 | 2·00 | — | — | 1·98 | 1·63 | 5 | 0·9 | — |
| MTX Day 0 only | $\bar{1}$·86 | 0·258 | 34 | 0·72 | — | 7 | 2·19 | 1·70 | 5 | — | — |
| Days 0+2 | $\bar{1}$·39 | 0·258 | 5 | 0·25 | <0·001 | 7 | 9·03 | 2·81 | 5 | 0·005 | 6 |
| Days 0+3 | $\bar{1}$·22 | 0·275 | 6 | 0·17 | <0·001 | 7 | 7·69 | 2·83 | 6 | 0·01 | 7 |
| Days 0+4 | $\bar{1}$·29 | 0·302 | 6 | 0·19 | <0·001 | 9 | 5·38 | 3·56 | 6 | 0·2 | 8 |
| Days 0+5 | $\bar{1}$·26 | 0·255 | 5 | 0·18 | <0·001 | 11 | 7·02 | 3·68 | 10 | 0·025 | 9 |
| Days 0+6 | <$\bar{1}$·00 | | 5 | 0·1 | <0·001 | 12 | 11·02 | 1·56 | 5 | <0·001 | 10 |
| | | (all values <$\bar{1}$·00) | | | | | | | | | |
| Days 0+7 | $\bar{1}$·36 | 0·364 | 11 | 0·23 | <0·001 | 13 | 2·20 | 2·53 | 10 | 0·99 | 11 |
| Days 0+8 | $\bar{1}$·88 | 0·157 | 4 | 0·76 | 0·9 | 15 | ND | — | — | — | 11–12 |
| Days 0+9 | $\bar{1}$·73 | 0·220 | 10 | 0·54 | 0·2 | 16 | 1·03 | 1·683 | 5 | 0·4 | 12 |
| Days 0+10 | 0·17 | 0·182 | 6 | 1·48 | 0·01 | 16 | ND | — | — | — | 12 |
| Days 0+11 | $\bar{1}$·91 | 0·281 | 5 | 0·81 | 0·7 | 18 | −2·34 | 3·40 | 5 | 0·05 | 16 |
| Days 0+12 | $\bar{1}$·90 | 0·239 | 5 | 0·79 | 0·8 | 18 | ND | — | — | — | 18 |
| Days 0+14 | $\bar{1}$·72 | 0·165 | 4 | 0·52 | 0·4 | 20 | ND | — | — | — | 19 |
| Days 0+21 | $\bar{1}$·93 | 0·07 | 5 | 0·85 | 0·6 | 28 | −1·20 | 5·51 | 5 | 0·3 | 26 |

* *vs.* Day 0 only.

The day upon which the lowest neutrophil count was recorded was generally 6 days after the last course of MTX. The lowest weights were generally observed 4 days after the last course of MTX.

Counts are shown as i.u. (× $10^9$/l) or $\log_{10}$ i.u.

ND—Not done. Initial weights not recorded.

separated by more than 6 days (i.e. on Days 7, 9, 11 or 14) the weight change is no longer significant. The lowest weights were generally recorded 4 days after the second course of MTX (see Table I). These calculations were made on the basis of the lowest recorded weight, compared with initial body weight, throughout the experiment, so daily fluctuations in body weight will account for minor weight losses.

The neutropenia data show a similar picture. When the second course of MTX is given on Days 2–7, there is statistically significant neutropenia compared with that produced by a single course of MTX. When the second course of MTX was given on Days 8–21, there was no significant depression of neutrophil counts com-

Fig. 3.—Correlations between bone marrow cellularity following a single course of MTX and maximum neutropenia following two courses of MTX at various intervals.

pared with that produced by a single course of MTX. When the second course was given on Day 10, there was significant *elevation* compared with a single course. The greatest neutropenia generally occurred 6 days after the second course of MTX (Table I).

When the level of neutropenia obtained at various intervals between courses is compared with bone marrow cell numbers/mg bone marrow (Fig. 3) it can be seen that increased neutropenia is associated with second courses of MTX at any time during the period when bone marrow numbers are reduced by the first course: *i.e.* superadditive effects on blood neutrophils occur, not only during recovery of bone marrow numbers, but also during the initial period when bone marrow numbers are falling.

The overshoot in neutrophil recovery described in relation to Fig. 2 is tabulated for all groups of rats in Table II. An interesting point arising out of this Table is that significant reactive neutrophilia occurs in double courses separated by 2–10 days, whereas superadditive neutropenia is only seen in courses separated by 2–7 days. The time of the maximum neutrophil count is shown in Table II, and was generally 9 days after the second course of MTX. In this respect it would appear that measurement of the late overshoot in neutrophil counts following second MTX courses is the most sensitive method we have found for detecting a superadditive effect.

*The effect of a third course of MTX, given 21 days after the previous course, on blood neutrophil counts*

The previous section showed that a 3-week interval between a first and second course of MTX resulted in identical changes in blood neutrophil counts after each course. To see if the increased neutrophil depression observed after double MTX courses would have a long-term effect on the susceptibility of the bone marrow myeloid cells to MTX, some of the groups of rats described in the previous section were subjected to a third course of MTX, 3 weeks after they had received their second course. The results are shown in Table III. These indicate that by 3 weeks recovery had occurred in all groups, and that a further course of MTX at that time produced the same pattern of neutropenia as that seen after a single course.

TABLE II.—*Mean Values for Late Reactive Neutrophilia*

| Group | Mean of $\log_{10}$ counts | ± s.d. | n | Geometric mean | *P* (*vs.* Day 0 only) | Day of greatest neutrophil count |
|---|---|---|---|---|---|---|
| Controls | 0·43 | 0·053 | 5 | 2·69 | 0·5 | — |
| MTX Day 0 only | 0·40 | 0·100 | 5 | 2·51 | — | 12 |
| Days 0+2 | 0·78 | 0·138 | 5 | 6·03 | 0·001 | 11 |
| Days 0+3 | 0·78 | 0·213 | 6 | 6·03 | 0·01 | 12 |
| Days 0+4 | 0·67 | 0·228 | 5 | 4·68 | 0·05 | 12 |
| Days 0+5 | 0·61 | 0·075 | 5 | 4·07 | 0·01 | 14 |
| Days 0+6 | 0·59 | 0·067 | 5 | 3·89 | 0·01 | 15 |
| Days 0+7 | 0·76 | 0·149 | 6 | 5·75 | 0·01 | 16 |
| Days 0+8 | 0·93 | 0·214 | 4 | 8·51 | 0·01 | 20 |
| Days 0+9 | 0·72 | 0·140 | 6 | 5·25 | 0·01 | 18 |
| Days 0+10 | 0·70 | 0·117 | 6 | 5·01 | 0·01 | 21 |
| Days 0+11 | 0·53 | 0·119 | 5 | 3·39 | 0·1 | 21 |
| Days 0+14 | 0·58 | 0·138 | 4 | 3·80 | 0·05 | 26 |

All rats, including the controls, were counted on each day. The figures in Columns 2 and 5 represent the means of the highest neutrophil counts recorded for each animal.

The day upon which the highest neutrophil count was recorded was generally 9 days following the last course of MTX. Counts recorded as i.u. ($\times 10^9$/l) or $\log_{10}$ i.u.

TABLE III.—*Maximum Neutropenia after a Third Course of Methotrexate*

| Group | Mean of $\log_{10}$ counts | ± s.d. | n | Geometric mean | *P* (*vs.* Day 0 only) |
|---|---|---|---|---|---|
| MTX Day 0 only | $\bar{1}$·91 | 0·20 | 5 | 0·81 | — |
| Days 0+21 | $\bar{1}$·93 | 0·07 | 5 | 0·85 | 0·9 |
| Days 0+2+21 | $\bar{1}$·74 | 0·25 | 5 | 0·55 | 0·3 |
| Days 0+3+24 | $\bar{1}$·94 | 0·16 | 6 | 0·87 | 0·9 |
| Days 0+4+25 | $\bar{1}$·68 | 0·31 | 5 | 0·48 | 0·2 |
| Days 0+5+26 | $\bar{1}$·84 | 0·08 | 5 | 0·69 | 0·7 |
| Days 0+6+27 | $\bar{1}$·75 | 0·26 | 5 | 0·56 | 0·4 |
| Days 0+7+28 | $\bar{1}$·87 | 0·39 | 5 | 0·74 | 0·8 |
| | *Pooling All Available Data* | | | | |
| MTX Day 0 only | $\bar{1}$·86 | 0·258 | 34 | 0·72 | — |
| All groups given MTX 21 days after previous course | $\bar{1}$·77 | 0·216 | 36 | 0·59 | 0·2 |

Counts are shown as i.u. ($\times 10^9$/l) or $\log_{10}$ i.u.

*The effect of large-dose courses of methotrexate*

When a large dose of MTX is given (0·1 mg/100 g body weight/injection) the results obtained are somewhat different. The major difference observed was that there were deaths associated with a second course of MTX given on Days 2–5 (Table IV). The greatest weight loss and mortality was seen when the second course of MTX was given on Day 4. The effects of moderate dose courses of MTX at this interval were relatively small in relation to loss of body weight (Table I).

The blood neutrophil data show that the most profound neutropenia occurs when the second course of MTX is given on Days 3 or 4 instead of Day 6. Because of the small numbers of rats used in this experiment, the neutropenia associated with a second course of MTX does not reach statistically significant levels when compared with a single course of MTX. The time of maximum neutropenia was also more variable, ranging from 4 days (0 + 2) to 9 days (0 + 9). However, all groups, including that given a single course of MTX, must be regarded as being at risk of infection. This may account for the mortalities occurring in this experiment. Pathological examination of rats showed anaemia with marked lung haemorrhages. In addition, histological examination of the lungs demonstrated bacterial infection. Macroscopic examination of the gastrointestinal tract did not reveal pathological changes and haemorrhage, although this has been reported as being associated with mortality at 4–5 days following a large single dose of MTX (Vitale *et al.*, 1954).

### *Blood and bone marrow leucocyte counts following i.p. injection of leukaemia cells*

Fig. 4 shows the $\log_{10}$ deviation from control of total white blood cells, blood neutrophils and bone marrow cells/mg when no MTX is given.

Fig. 4.—The effects on bone marrow cell numbers, total white blood cell numbers and blood neutrophil numbers of an i.p. inoculation of $10^7$ NDM 10 cells. By 17 days after the inoculation the tumour cells, 5/9 rats had died. See Table V for details of survival.

These values are within normal limits up to Day 7, when the blood counts become elevated. This elevation is due to both the appearance of leukaemic cells in the blood, and to a marked rise in granulocytes. Between Days 9 and 14, the number of bone marrow cells per mg weight of bone marrow becomes significantly depleted. Thus, it seems reasonable to assume that the rapid increase in blood neutrophil numbers is mainly due to release of cells from the bone marrow. The trigger for this release has not been determined. The total white blood cell count increases until the death of the animal, when it has reached levels of 200–600 × $10^9$/l. The neutrophil count is elevated up to Day 12, when there is a reversal, and the count rapidly decreases; and at death the neutrophil count is very low when, because of the extremely high total count, neutrophil numbers are difficult to estimate accurately.

### *The effect of MTX on the progress of transplanted leukaemia*

When MTX is administered, the onset of leukaemia, as determined by the time at which the total white blood cell count reaches 30 × $10^9$/l, is delayed, and survival is extended. An example of a typical experiment is shown in Fig. 5. The onset of leukaemia is extended from Day 9 to

Fig. 5.—An example of the delay in the onset of leukaemia and extension of survival time due to the administration of two courses of MTX separated by 3 days. The onset of leukaemia was delayed by 7 days, and the survival time was extended by 10 days.

TABLE IV.—*Mean Values for Neutropenia and Weight Loss, and Deaths recorded in High-dose Experiments*

| | Mean lowest observed neutrophil counts | | | | | | Mean maximum depression of body weight | | | | | |
|---|---|---|---|---|---|---|---|---|---|---|---|---|
| Group | Mean of $\log_{10}$ counts | ± s.d. | n | Geometric mean | $P$* | Day of lowest neutrophil count | % weight loss | ± s.d. | n | $P$* | Day of lowest weight | Deaths |
| MTX Day 0 only | $\bar{1}$·49 | 0·34 | 5 | 0·38 | — | 6 | 3·70 | 1·80 | 10 | — | — | 0/10 |
| Days 0+2 | $\bar{1}$·23 | 0·40 | 3 | 0·23 | 0·5 | 6–7 (Died) | 14·25 | 3·08 | 9 | <0·001 | 6–7 (Died) | 3/9 |
| Days 0+3 | <$\bar{1}$·00 | (all values <$\bar{1}$·00) | 3 | <0·10 | — | 6–7 | 12·09 | 10·72 | 8 | 0·05 | 7–9 | 2/8 |
| Days 0+4 | $\bar{1}$·10 | 0·17 | 3 | 0·13 | 0·2 | 7–10 | 17·41 | 5·14 | 8 | <0·001 | 8–12 | 6/8 |
| Days 0+5 | $\bar{1}$·26 | 0·24 | 3 | 0·20 | 0·4 | 7–10 | 6·28 | 6·27 | 9 | 0·3 | 9–11 | 1/9 |
| Days 0+7 | $\bar{1}$·37 | 0·32 | 3 | 0·27 | 0·7 | 10 | 6·11 | 5·77 | 8 | 0·3 | 11 & 15 | 0/8 |
| Days 0+8 | $\bar{1}$·44 | 0·43 | 3 | 0·37 | 0·9 | 14 | 5·53 | 1·14 | 3 | 0·2 | 11 | 0/3 |
| Days 0+9 | $\bar{1}$·65 | 0·33 | 3 | 0·53 | 0·6 | 18 | 1·59 | 1·98 | 3 | 0·2 | 15 | 0/3 |

* *vs.* Day 0 only.

The differences in numbers of rats studied in the two sections of the Table represent rats that were treated but not subjected to daily anaesthesia and white cell counts.

The day of the lowest recorded weight is in some instances the day upon which rats died.

Counts are shown as i.u. (× $10^9$/l) or $\log_{10}$ i.u.

TABLE V.—*The Onset of Leukaemia and Survival with Different MTX Schedules*

| | Onset of leukaemia (Days) | | | | | Median | Survival (Days) | | | | | Median |
|---|---|---|---|---|---|---|---|---|---|---|---|---|
| *Experiment* 1 | | | | | | | | | | | | |
| No MTX | 8 | 8 | 9 | 9 | 10 | 9 | 13 | 14 | 14 | 14 | 15 | 14 |
| MTX Day 0 only | 13 | 13 | 13 | 14 | 14 | 13 | 19 | 19 | 20 | 20 | 21 | 20 |
| Days 0+2 | 13 | 14 | 14 | 14 | 15 | 14 | 20 | 21 | 23 | 23 | 24 | 23 |
| Days 0+3 | 15 | 15 | 15 | 16 | 17 | 15 | 23 | 23 | 24 | 24 | 26 | 23 |
| Days 0+4 | 12 | 14 | 15 | 16 | | 14·5 | 21 | 22 | 23 | 24 | | 22·5 |
| Days 0+5 | 13 | 13 | 13 | 14 | 15 | 13 | 20 | 21 | 21 | 22 | 23 | 21 |
| Days 0+6 | 14 | 14 | 15 | 16 | | 14·5 | 22 | 22 | 22 | 23 | | 22 |
| *Experiment* 2 | | | | | | | | | | | | |
| No MTX | 8 | 9 | 9 | 9 | | 9 | 18 | 19 | 19 | 19 | | 19 |
| MTX Days 0+3 | 15 | 15 | 16 | 17 | 21 | 16 | 24 | 24 | 25 | 25 | 27 | 25 |
| Days 0+7 | 14 | 15 | 16 | 16 | 17 | 16 | 22 | 22 | 23 | 24 | 25 | 23 |
| Days 0+9 | 12 | 12 | 12 | 12 | 20 | 12 | 22 | 23 | 23 | 25 | 26 | 23 |
| Days 0+12 | 13 | 13 | 13 | 14 | 14 | 13 | 20 | 23 | 23 | 24 | 25 | 23 |

Day 16, and the survival from Day 14 to Day 24. Also shown in Fig. 5 is the fact that the neutrophil count is still reduced on Day 9 after a single course of MTX, which is presumably due to the myelotoxicity of MTX described previously.

The onset of leukaemia, and survival times for other MTX schedules is shown in Table V. Table VI shows the statistical significance (in terms of probability, $P$) of the results in Table V. In Experiment 1, the onset of leukaemia is significantly delayed by a single course of MTX, but significant further delay was not achieved by additional courses of MTX, except when the second course was given at Day 3. The administration of a second course of MTX significantly extends survival in all cases, except where the courses were separated by 5 days. In the second experiment, course intervals of 7, 9 and 12 days are compared with MTX on Days 0 and 3. MTX on Days 0 and 12 is associated with earlier onset of leukaemia. However, the spacing of courses of MTX, within the limits of these experiments, does not appear to be critical in terms of survival.

Thus, MTX given in two courses separated from 2 to 12 days is equally effective in this respect. However, the onset of leukaemic phase appears to be most effectively delayed by MTX on Days 0 and 3.

### DISCUSSION

Vogler *et al.* (1973) showed similar bone marrow kinetics in mice after a single dose of MTX. They found that a single

TABLE VI.—*Statistical Analysis (Probabilities) of the Onset of Leukaemia and Survival Time (Data in Table V)*

*Experiment* 1

| | Time to reach $30 \times 10^3$ WBC *vs.* MTX Day 0 only | Survival *vs.* MTX Day 0 only | Survival *vs.* MTX Days 0+3 |
|---|---|---|---|
| No MTX | 0·01 | 0·02 | Not analysed |
| MTX Day 0 only | — | — | 0·02 |
| MTX Days 0+2 | 0·3 | 0·05 | 0·3 |
| Days 0+3 | 0·02 | 0·02 | — |
| Days 0+4 | 0·2 | 0·05 | 0·3 |
| Days 0+5 | 0·99 | 0·1 | 0·2 |
| Days 0+6 | 0·1 | 0·02 | 0·05 |

*Experiment* 2

| | Time to reach $30 \times 10^3$ WBC *vs.* MTX Days 0+3 | Survival *vs.* MTX Days 0+3 |
|---|---|---|
| No MTX | 0·01 | 0·02 |
| MTX Days 0+3 | — | — |
| Days 0+7 | 0·7 | 0·1 |
| Days 0+9 | 0·1 | 0·3 |
| Days 0+12 | 0·02 | 0·1 |

dose of 60 mg/kg caused a reduction in the proliferating bone marrow pool to 43% of control on Day 2, which returned to initial levels by Day 6 (*cf.* Fig. 1). When they repeated the dose of MTX on Day 3, at a time when they had demonstrated that the number of colony-forming cells was elevated, there was a fall in the proliferating pool to 8% of the control value. This result compares well with our results showing that large-dose courses of MTX at 3- or 4-day intervals resulted in maximal neutropenia, whereas neutropenia when the second course was given at Day 8 did not result in greater neutropenia than seen after a single course of the drug (Table IV).

Following a second dose on Day 3, Vogler *et al.* (1973) demonstrated a ninefold increase in colony-forming cells on Day 5. This may be analogous to our results showing marked polymorphonuclear leucocytosis in rats given moderate dose treatment with the second course given on Days 2–4 (Table III). Although the numbers of rats surviving in the high-dose experiments were too small to allow a confident statement about the degree of reactive neutrophil leucocytes, it is interesting to note that neutrophil counts above $40 \times 10^9/l$ were recorded in 2 rats surviving after second courses on Days 3 and 4. This hints that the degree of overshoot may relate to the degree of neutropenia induced. Such a conclusion was drawn by Morley *et al.* (1971), who showed that colony-stimulating factor levels in irradiated mice were directly proportional to the degree of neutropenia induced. Such a clear-cut relationship, however, is not supported by the fact that reactive polymorphonuclear leucocytes occurred in rats given second doses of drug between 8 and 10 days when no superadditive neutropenia was seen. In fact, the overshoot of neutrophil counts after second courses on Days 8 and 9 were higher than any recorded following the shorter-spaced double doses which caused most marked neutropenia.

If one considers the bone marrow studies, there is a close correlation between the degree of subsequent neutropenia and the reduction in bone marrow cell numbers at the time of the second course of MTX (Fig. 3). It is impossible, from our studies, to draw any conclusions about the rate of division of single cell types within the bone marrow, and such data might correlate more closely with the neutropenia induced by a second course of MTX.

However, it is clear that MTX has its effects on the myelocyte, which has a maturation time of some 5–6 days before appearing in the blood as a neutrophil, rather than on the stem cell, which would not show an effect for 10–12 days (van Furth, 1974).

Thus there is a period of some 5–6 days during which the normal myelocyte population is re-formed, and during which few neutrophils appear in the blood. This may be seen in Fig. 1. When cells are lost from the myelocyte pool, a significant number of precursor cells become myelocytes (Craddock, 1973). It may be that superadditive effects in part relate to the length of time and the amount of MTX which persists in myeloid precursors, as well as to the rate of proliferation by these cells. We have performed experiments to assess the effect of MTX on rats undergoing increased neutrophil production as a result of experimentally induced pyelonephritis. These studies indicated that there was not a simple correlation between MTX-induced neutropenia and the rate of production of neutrophils (Harding and MacLennan, to be published).

Finally, one may ask what are the points one can gain from this study which may be of help in minimizing myelotoxicity following multiple MTX courses in man? Clearly the persistency of superadditive toxicity will vary with the dose given and the length of courses. Also the dose response in man is unlikely to be the same as in rats. However, it is reasonable to conclude that superadditive toxic effects of repeat doses of MTX can be avoided by increasing the spacing between courses: a day or two one way or the other can make a great deal of difference. For example, the severe superadditive neutropenia occurs when courses are separated by 7 days, while the additive effect is negligible when 8 days are allowed to elapse between courses. It is clear that toxicity cannot be avoided by reducing the spacing between courses, so that a second dose is given when bone marrow proliferation is most depressed.

The data presented here are compatible with the conclusion that the marked myelotoxicity of the Medical Research Council's UKALL I trial related to the close spacing of 5-day courses of MTX (M.R.C. Working Party, 1975). The relative lack of myelotoxicity in UKALL II, where identical courses of MTX are separated by 3 weeks (M.R.C. Working Party, 1976) again accords with our data.

For our studies of the effects on the antileukaemic activity of altering the interval between courses of MTX, we have used as our model of acute lymphoblastic leukaemia (ALL) a rat T-cell leukaemia which has been described as having some similar pathophysiological features to human ALL (Dibley *et al.*, 1976). However, in terms of cell division times and the proportion of cells in cycle, this rat leukaemia is different from human ALL. The rat leukaemia has a particularly rapid doubling time of 1 day (unpublished results), with a survival time of only 2–3 weeks following i.p. injection of $10^7$ leukaemic lymphoblasts. In human ALL the leukaemic cells are not as rapidly dividing, and undergo division at a rate similar to or less than normal cells in the bone marrow: the mean generation time in ALL patients has been estimated as being 2–8 days (Clarkson, 1969). Thus it is possible that MTX, which principally affects active or dividing cells, would have a different anti-leukaemic effect in patients from that in the rats of our studies. However, leukaemic cells proliferate faster when many cells have been killed by therapy and their concentration has been reduced (Clarkson, 1969). Hryniuk (1972) showed that the anti-leukaemic effects of MTX are proportional to the rate of cell proliferation. On these grounds MTX is more likely to be an effective drug in the maintenance of remission, which is the situation we have simulated in our experiments. Skipper *et al.* (1957) showed a correlation between inoculum size and curability with MTX, under favourable conditions. It was suggested that this was due to the mutation rate of leukaemic populations resulting in MTX resistance. Although the anti-leukaemic effects we have observed are similar over a range of treatment schedules, the best long-term schedule would be one based on minimal host toxicity. Goldin *et al.* (1956) showed in mice that the best schedule for early therapy is infrequent heavy doses of MTX (in his studies, every 4 days rather than daily). The same authors (Goldin *et al.*, 1954*a*) had earlier shown that the greatest anti-leukaemic effects were obtained with multi-dose schedules (Days 2 + 4 + 6). However, these were achieved at the cost of high mortality from host toxicity. They overcame this problem by using even higher doses of MTX, with administration of Citrovorum factor 12 h after the administration of MTX (Goldin *et al.*, 1954*b*). Tattersall *et al.* (1975) reduced MTX host toxicity by thrice daily administration of thymidine, without inhibiting the anti-tumour effects. In our present study, blood neutrophils are depressed to an extent greater than that caused by a single course of MTX if a second course of MTX is given on Days 2 to 7, but not if given after 8 or more days. We also show that body weight is depressed if the second course of MTX is given on Days 2 to 6, but not if given later. However, the anti-leukaemic effects of MTX courses separated by 2 to 12 days are similar, and thus the courses could be spaced to give optimum anti-leukaemic effects with minimal host toxicity.

### REFERENCES

Acute Leukaemia Group B (1961) Studies of Sequential and Combination Antimetabolite Therapy in Acute Leukaemia: 6-mercaptopurine and Methotrexate. *Blood*, **18**, 431.

ACUTE LEUKAEMIA GROUP B (1969) Acute Lymphocytic Leukaemia in Children. Maintenance Therapy with Methotrexate Administered Intermittently. *J. Am. med. Ass.*, **207**, 923.

AUR, R. J. A. & PINKEL, D. (1972) Total Therapy of Acute Lymphocytic Leukaemia. *Cancer, N.Y.*, **29**, 381.

BERLIN, N. I., RALL, D., MEAD, J. A. R., FREIREICH, E. J., VAN SCUTT, E., HERTZ, R. & LIPSETT, M. B. (1963) Folic Acid Antagonists. *Ann. intern. Med.*, **59**, 931

BERTINO, J. R., HILLCOAT, B. C. & JOHNS D. G. (1967) Folate Antagonists: Some Biochemical and Pharmacological Considerations. *Fed. Proc.*, **26**, 893.

BLAKLEY, R. L. (1969) *The Biochemistry of Folic Acid and Related Pteridines.* Amsterdam: North Holland.

BORSA, J. & WHITMORE, G. F. (1969) Cell Killing Studies on the Mode of Action of Methotrexate on L Cells. *Cancer Res.*, **29**, 737.

CLARKSON, B. D. (1969) Review of Recent Studies of Cellular Proliferation in Acute Leukaemia. *Natl Cancer Inst. Monogr.*, **30**, 81.

CRADDOCK, CH. A. (1973) Granulocyte Kinetics. In: *Haematology.* Eds. W. J. Williams, E. Beutler, A. J. Erslev and R. W. Rundles. New York: McGraw Hill.

DELMONTE, L. & JUKES, T. H. (1962) Folic Acid Antagonists in Cancer Chemotherapy. *Pharmacol. Rev.*, **14**, 91.

DIBLEY, M., DORSCH, S. & ROSER, B. (1975) T Cell Leukaemia in the Rat: Pathophysiology. *Pathology*, **7**, 219.

GALTON, D. A. G. (1969) In *Proceedings of VIIIth International Congress of Haematology.* Tokyo: Pan Pacific Press, p. 467.

GOLDIN, A., MANTEL, N., GREENHOUSE, S. W., VENDITTI, J. M. & HUMPHREYS, S. R. (1954*a*) Factors Influencing the Specificity of Action of an Anti-leukaemic Agent (Aminopterin). Time of Treatment and Dosage Schedule. *Cancer Res.*, **14**, 311.

GOLDIN, A., VENDITTI, J. M., HUMPHREYS, S. R., DENNIS, D., MANTEL, N. & GREENHOUSE, S. W. (1954*b*) Factors Influencing the Specificity of Action of an Anti-leukaemic Agent (Aminopterin). Multiple Treatment Schedules plus Delayed Administration of Atrovorin Factor. *Cancer Res.*, **15**, 56.

GOLDIN, A., VENDITTI, J. M., HUMPHREYS, S. R. & MANTEL, N. (1956) Modification of Treatment Schedules in the Management of Advanced Mouse Leukaemia with Amethopterin. *J. natn. Canc. Inst.*, **17**, 203.

HRYNIUK, W. M. (1972) Purineless Death as a Link between Growth Rate and Cytotoxicity by Methotrexate. *Cancer Res.*, **32**, 1506.

KRIVIT, W., BRUBAKER, C., THATCHER, L. G., PIERCE, M., PERRIN, E. & HARTMANN, J. R. (1968) Maintenance Therapy in Acute Leukaemia of Childhood. *Cancer, N.Y.*, **21**, 352.

MORLEY, A., RICKARD, K. A., HOWARD, D. & STOHLMAN, F. (1971) Studies on the Regulation of Granulopoiesis. IV—Possible Humoral Regulation. *Blood*, **37**, 14.

NAGAO, T., LAMPKIN, B. C. & MAUER, A. M. (1970) Maintenance Therapy in Acute Childhood Leukaemia. *J. Pediatr.*, **76**, 134.

SAUNDERS, E. F., KAUDER, E. & MAUER, A. M. (1972) Sequential Therapy of Acute Leukaemia in Childhood. *J. Pediatr.*, **70**, 632.

SKIPPER, H. E., SCHABEL, F. M., BELL, M., THOMSON, J. R. & JOHNSON, S. (1957) On the Curability of Experimental Neoplasms. I—Amethopterin and Mouse Leukaemias. *Cancer Res.*, **17**, 717.

TATTERSALL, M. H. N., BROWN, B. & FREI, E. (1975) The Reversal of Methotrexate Toxicity by Thymidine with Maintenance of Anti-tumour Effects. *Nature, Lond.*, **253**, 198.

VAN FURTH, R. (1974) The Kinetics and Functions of Polymorphonuclear and Mononuclear Phagocytes. *Prog. Immunol. II.*, **4**, 73.

VITALE, J. J., ZAMCHECK, N., DI GIOGIO, J. & HEGSTED, D. M. (1954) Effects of Aminopterin Administration on the Respiration and Morphology of the Gastrointestinal Mucosa of Rats. *J. Lab. clin. Med.*, **43**, 583.

VOGLER, W. R., MINGIOLI, E. S. & GARWOOD, F. A. (1973) The Effect of Methotrexate on Granulocytic Stem Cells and Granulopoiesis. *Cancer Res.*, **33**, 1628.

WORKING PARTY ON LEUKAEMIA IN CHILDHOOD (1975) Analysis of Treatment in Childhood Leukaemia. I—Predisposition to Methotrexate Induced Neutropenia after Craniospinal Irradiation. *Br. med. J.*, ii, 563.

WORKING PARTY ON LEUKAEMIA IN CHILDHOOD (1976) Analysis of Treatment in Childhood Leukaemia. II—Timing and the Toxicity of Combined 6 Mercaptopurine and Methotrexate Maintenance Therapy. *Br. J. Haematol.*, **33**, 179.